# Bridging silicone and metallic stents for tracheobronchial stenosis in relapsing polychondritis: A case report

**DOI:** 10.1097/MD.0000000000043807

**Published:** 2025-08-15

**Authors:** Lingyun Zhang, Zhigang Hu, Wenxin Li, Yuqin Li, Qiaoyu Yang, Bijun Zhao, Fanjun Zeng, Long Lin, Xinyun Song

**Affiliations:** a Department of Respiratory and Critical Care Medicine, Yichang Central People's Hospital, The First Clinical Medical College of China Three Gorges University, Yichang, China; b Department of Respiratory and Critical Care Medicine, Yichang Second People's Hospital, Yichang, China.

**Keywords:** airway stenosis, metallic stent, polychondritis, silicone stent

## Abstract

**Rationale:**

Relapsing polychondritis is a rare autoimmune disease that commonly leads to tracheobronchial stenosis, presenting significant treatment challenges.

**Patient concerns:**

This case report describes a 64-year-old male with relapsing polychondritis who developed severe tracheobronchial stenosis.

**Diagnoses:**

Computed tomography scanning revealed stenosis of the trachea and bronchi, while bronchoscopy confirmed occlusion or stenosis.

**Interventions:**

Initially, the patient presented with chest pain, fever, and dyspnea. After treatment with corticosteroids and immunosuppressants, fever and chest pain improved, though dyspnea persisted. A silicone stent was inserted, leading to a significant improvement in dyspnea. However, due to granulation tissue hyperplasia at the lower edge of the left main bronchus stent, a metallic stent was used to bridge with the silicone stent 4 years later.

**Outcomes:**

The patient has been followed for 7 years with stable disease control.

**Lessons:**

For complex airway stenosis caused by relapsing polychondritis, combined placement of silicone stent and metal stent may be considered as a therapeutic option.

## 1. Introduction

Relapsing polychondritis (RP) is a rare systemic autoimmune disease characterized by inflammation and destruction of cartilage. Approximately 50% of patients with RP experience airway involvement, which leads to tracheobronchial stenosis and can result in life-threatening respiratory complications.^[1]^ Current treatments, including corticosteroids and immunosuppressants, often fail to address the structural damage to the airway. Airway stenting, particularly with silicone stents, has been used to provide mechanical support, though complications such as granulation tissue hyperplasia remain a challenge. Silicone stents effectively support the stenotic airway, improving dyspnea symptoms.^[2,3]^ To our knowledge, no prior studies have reported the combined use of silicone and metallic stents for tracheobronchial stenosis associated with RP. This case report presents a novel treatment strategy and discusses its potential advantages and limitations.

## 2. Case report

A 64-year-old male farmer, previously healthy with no history of hypertension, diabetes, or coronary heart disease, began experiencing chest pain in May 2017. The pain worsened and was accompanied by intermittent fever (up to 39.1 °C), mild cough, white sputum, chest tightness, dyspnea, and weight loss. He was initially diagnosed with pleurisy at a local hospital, but his symptoms did not improve despite anti-infective, cough-suppressive, and asthma-relieving treatments. He was admitted to our hospital on August 14, 2017.

**Figure 1.**
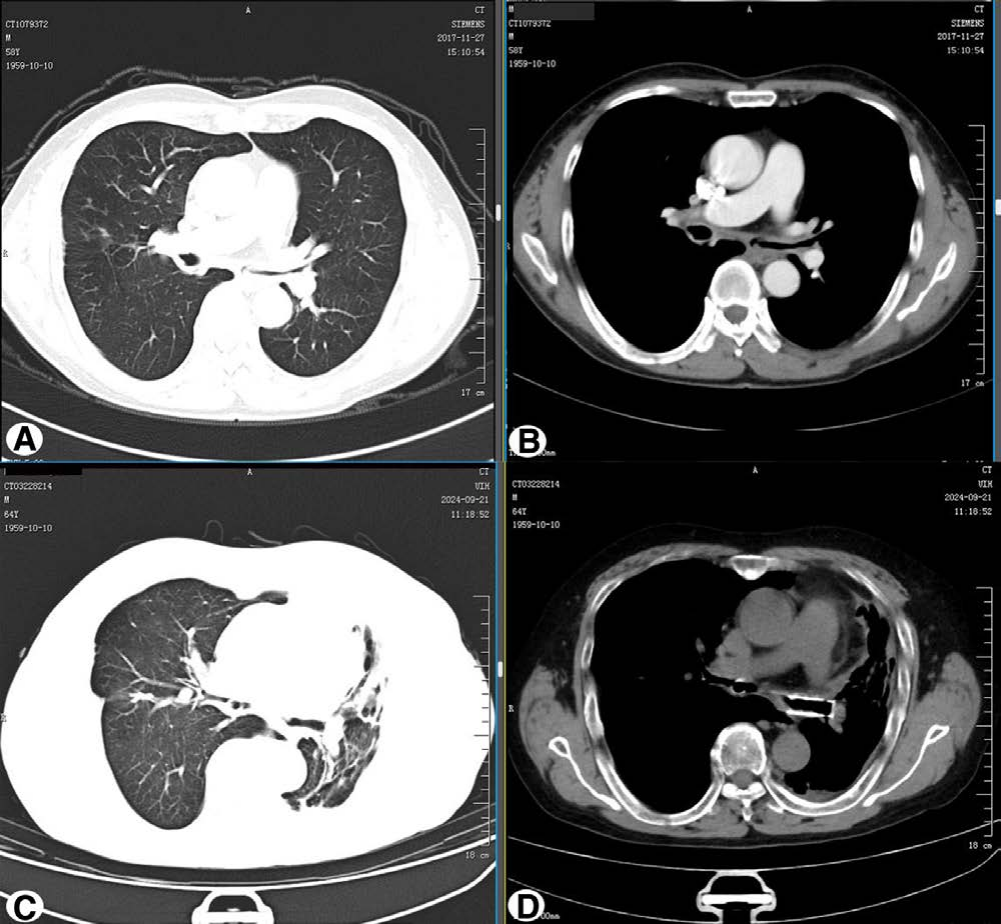
(A and B) Chest CT showed thickening of the tracheal and bronchial walls with narrowing of the lumen. (C and D) Chest CT showed atelectasis and bronchiectasis of the left lung, with chronic infection. CT = computed tomography.

**Figure 2.**
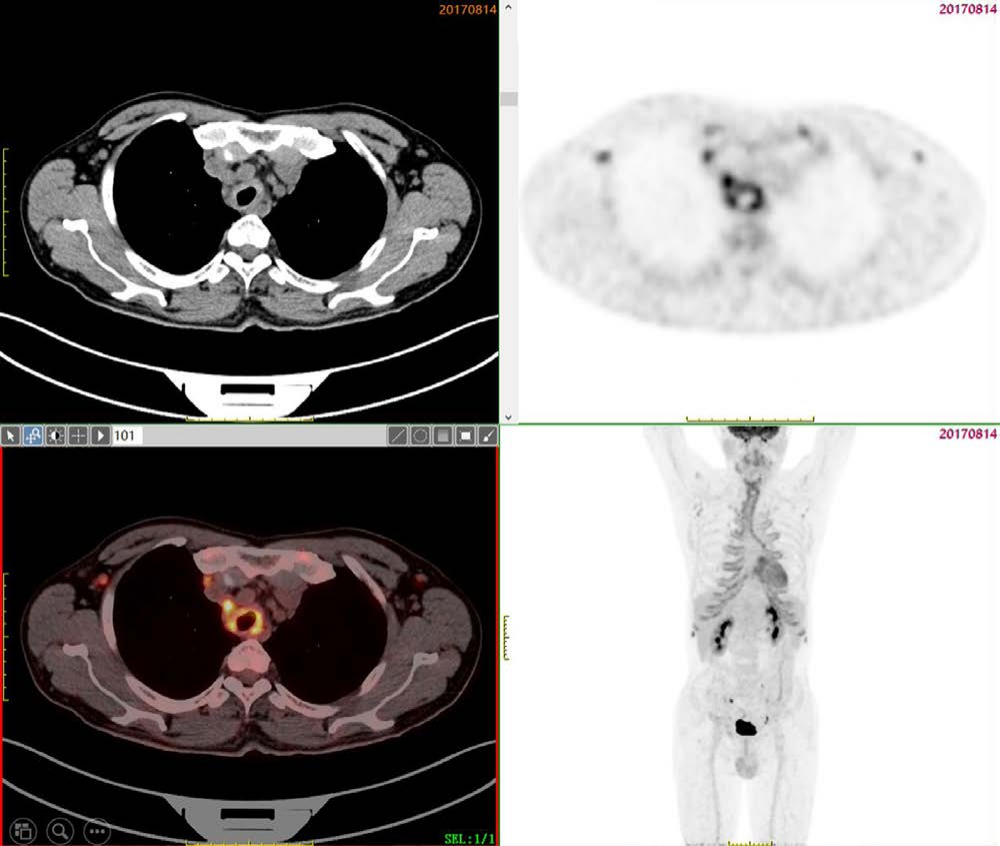
PET-CT demonstrated abnormal metabolic activity in the thyroid cartilage, tracheal cartilage, and bilateral rib cartilage. PET-CT = positron emission tomography–computed tomography.

**Figure 3.**
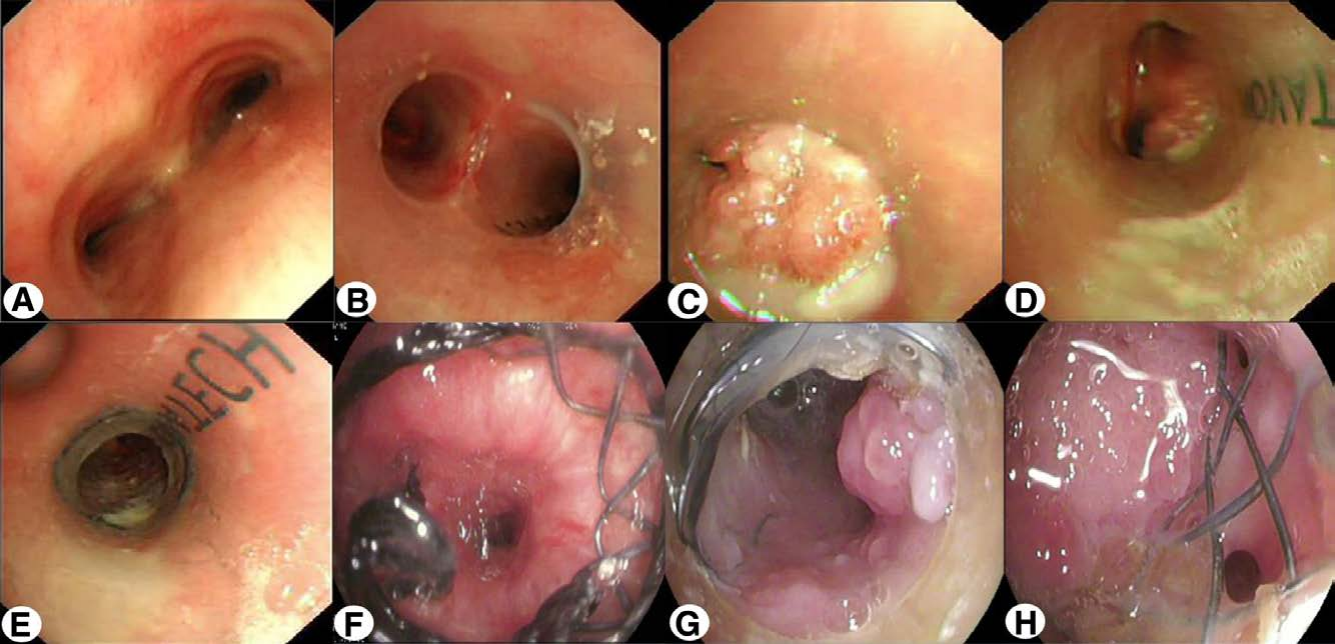
(A) Bronchoscopy showed dynamic stenosis of the lumen, most prominent at the end of exhalation. (B) Postoperative Y-shaped silicone stent placement. (C) Bronchoscopy revealed severe granulation tissue hyperplasia at the left main bronchus with almost complete stenosis of the lumen. (D) There is a minimal amount of granulation tissue hyperplasia at the lower edge of the right main bronchus. (E and F) Postoperative left main metallic stent bridging the silicone stent. (G and H) The left main bronchus metallic stent is twisted and deformed, with parts embedded in the wall.

On initial admission, the patient’s vital signs were: temperature 36.4 °C, pulse 86 beats per minute, respiration rate 26 breaths per minute, blood pressure 107/80 mm Hg, and oxygen saturation 94% on room air. Physical examination revealed tachypnea, no cyanosis or deformity of the ears and nose, and a barrel-shaped chest. Local tenderness was noted in the anterior sternal region. Breath sounds were slightly decreased in both lungs, without rales. The cardiac, abdominal, and neurological examinations were unremarkable. Laboratory tests showed normal blood routine, extractable nuclear antigen, anti-neutrophil cytoplasmic antibody, and rheumatoid factor. C-reactive protein was 38 mg/L, procalcitonin 0.09 ng/mL, and erythrocyte sedimentation rate 30 mm/h. Arterial blood gas analysis showed pH 7.35, partial pressure of CO_2_ 43.2 mm Hg, and partial pressure of O_2_ 63.1 mm Hg. Chest computed tomography (CT) showed thickening of the tracheal and bronchial walls with narrowing of the lumen (Fig. 1A and B). Pulmonary function tests revealed severe mixed ventilatory dysfunction with an obstructive pattern, and bronchoscopy showed dynamic stenosis of the lumen, most prominent at the end of exhalation (Fig. 3A). Positron emission tomography-CT (PET-CT) demonstrated abnormal metabolic activity in the thyroid cartilage, tracheal cartilage, and bilateral rib cartilage (Fig. 2).

On August 19, 2017, after multidisciplinary team discussion with the rheumatology and immunology department, the patient was diagnosed with RP and treated with prednisone (30 mg, qd), cyclophosphamide, and methotrexate. His chest pain and fever improved, and he was discharged, but his chest tightness, shortness of breath, and poor exercise tolerance persisted. On October 8, 2017, his condition worsened, and he returned to our hospital again. Despite symptomatic treatment, his dyspnea did not improve. With the consent of the patient and his family, on October 11, 2017, an airway Y-shaped silicone stent (Novatech, France, main body 16 mm × 60 mm, left main 13 mm × 30 mm, right main 13 mm × 20 mm) was placed under general anesthesia, which significantly relieved his dyspnea. Postoperative bronchoscopy showed the stent in good position (Fig. 3B). During follow-up, granulation tissue hyperplasia was observed at the lower edge of the left main bronchus stent, leading to recurrent obstruction. On August 1, 2020, bronchoscopy revealed severe granulation tissue hyperplasia at the left main bronchus with almost complete stenosis of the lumen (Fig. 3C), and a minimal amount of granulation tissue hyperplasia at the lower edge of the right main bronchus (Fig. 3D). Considering the patient’s critical condition and poor effect of repeated interventional treatment to remove polyps, a metallic covered stent (12 mm × 30 mm) was bridged with the silicone stent on August 6, 2020 (Fig. 3E and F), resulting in slight improvement in dyspnea. Subsequent bronchoscopic interventions were required to remove granulation tissue. By September 21, 2024, the metallic stent was found to be twisted and deformed, with part of it embedded in the bronchial wall (Fig. 3G and H). Chest CT showed atelectasis and bronchiectasis of the left lung, with chronic infection (Fig. 1C and D). The patient has been followed up to the present, and the condition is relatively stable.

## 3. Discussion

Although RP is rare, a significant proportion of affected patients experience involvement of the large airways. Upper airway stenosis in RP shares clinical features with other benign causes of airway obstruction, such as retrosternal goiter, which can cause fixed obstruction on pulmonary function tests.^[4]^ A retrospective study found that 55% of RP patients exhibit respiratory involvement, which is associated with significant functional impairment and higher mortality. Early diagnosis and treatment significantly improve prognosis.^[5]^ Both CT and PET-CT are critical for diagnosing RP, with PET-CT offering higher specificity and sensitivity, which can assist in targeted biopsy of affected areas and significantly increase the positive rate of biopsy.^[6]^ In this case, PET-CT showed widespread involvement of tracheal and rib cartilage. Although there were no classic signs of “saddle nose” or auricular chondritis, the patient met the Damiani and Levine criteria for RP diagnosis due to his fever and chest pain, which improved with corticosteroid treatment.^[7]^

Current treatment for RP primarily involve corticosteroids and immunosuppressants. Recently, some new biological agents, such as infliximab and tocilizumab, have been shown to be effective in some cases.^[8]^ However, in this patient, dyspnea persisted despite these treatments, likely due to irreversible damage to tracheal cartilage. For such patients, in addition to conservative treatment with internal medicine drugs, tracheostomy, T-tube placement, and airway stent placement are all available options.

Airway stent placement is a minimally invasive procedure that can quickly alleviate airway obstruction in patients. For benign dynamic airway stenosis requiring long-term management, silicone stents offer several advantages over metallic stents. These include easier customization, simpler removal, reduced risk of granulation tissue hyperplasia, and stable support strength.^[9]^ In this case, the left main bronchus stenosis was more severe at the onset of the disease. However, the patient did not undergo regular bronchoscopy after silicone stent placement, which led to significant granulation tissue hyperplasia and near-total obstruction of the left main bronchus. When the obstruction could no longer be effectively relieved, a metal covered stent was placed as a bridging solution. While metal covered stents can be used in such cases, they are generally not the first choice for benign bronchial stenosis due to their susceptibility to twisting, deformation, fatigue fractures, excessive granulation tissue formation, and difficulties with removal.

In conclusion, for patients with RP-related dynamic airway stenosis, silicone stents can effectively improve symptoms, though long-term complications such as granulation tissue hyperplasia and airway obstruction require careful management. The timing of stent intervention remains a critical issue, warranting further research.^[10]^ This case offers novel insights for clinicians in rheumatology and respiratory intervention, and has important clinical significance.

## Acknowledgments

The authors wish to thank the patient and medical staff of the department of pulmonary and critical care medicine for their help and collaborations.

## Author contributions

**Methodology:** Long Lin.

**Supervision:** Wenxin Li, Yuqin Li, Bijun Zhao, Fanjun Zeng, Xinyun Song.

**Writing – original draft:** Lingyun Zhang.

**Writing – review & editing:** Zhigang Hu, Yuqin Li, Qiaoyu Yang.
